# Immobilization and property of penicillin G acylase on amino functionalized magnetic Ni_0.3_Mg_0.4_Zn_0.3_Fe_2_O_4_ nanoparticles prepared *via* the rapid combustion process

**DOI:** 10.3389/fbioe.2023.1108820

**Published:** 2023-03-13

**Authors:** Mingyi Ma, Xiu Chen, Yao Yue, Jie Wang, Dawei He, Ruijiang Liu

**Affiliations:** ^1^ School of Pharmacy, Jiangsu University, Zhenjiang, China; ^2^ The People’s Hospital of Danyang, Affiliated Danyang Hospital of Nantong University, Zhenjiang, China; ^3^ Affiliated Kunshan Hospital, Jiangsu University, Suzhou, China

**Keywords:** magnetic Ni_0.3_Mg_0.4_Zn_0.3_Fe_2_O_4_ nanoparticles, immobilization, penicillin G acylase, reusability, surface modification

## Abstract

Penicillin G acylase plays an important role in the biocatalytic process of semi-synthetic penicillin. In order to overcome the disadvantages of free enzymes and improve the catalytic performance of enzymes, it is a new method to immobilize enzymes on carrier materials. And magnetic materials have the characteristics of easy separation. In the present study, the Magnetic Ni_0.3_Mg_0.4_Zn_0.3_Fe_2_O_4_ nanoparticles were successfully prepared by a rapid-combustion method and calcined at 400°C for 2 h. The surface of the nanoparticles was modified with sodium silicate hydrate, and the PGA was covalently bound to the carrier particles through the cross-linking of glutaraldehyde. The results showed that the activity of immobilized PGA reached 7121.00 U/g. The optimum pH for immobilized PGA was 8 and the optimum temperature was 45°C, the immobilized PGA exhibited higher stability against changes in pH and temperature. The Michaelis–Menten constant (K_m_) values of the free and immobilized PGA were 0.00387 and 0.0101 mol/L and the maximum rate (V_max_) values were 0.387 and 0.129 μmol/min. Besides, the immobilized PGA revealed excellent cycling performance. The immobilization strategy presented PGA had the advantages of reuse, good stability, cost saving and had considerable practical significance for the commercial application of PGA.

## Introduction

Beta-lactam antibiotics with distinguished anti-infective properties are widely used in medicine and healthcare (Zhang Y. M. et al., 2022; Zhang Y. F. et al., 2022; Zhou et al., 2022). Natural sources of antibiotics are no longer sufficient to meet human demand, so scientists use traditional chemical synthesis methods to obtain antibiotics. However, with the growing requirement for green products, traditional chemical synthesis methods have been replaced by emerging biological transformation processes. The 6-aminopenicillins acid (6-APA), which is the crucial raw material of semisynthetic β-lactam antibiotics (SSBA), is obtained through the hydrolysis reaction with penicillin G acylase as the enzyme catalysis (Cano-Cabrera et al., 2021; Wang et al., 2022). PGA plays an essential role in the synthesis of 6-APA. To improve its efficiency, the immobilization of PGA has received widespread attention (Li et al., 2020).

Penicillin G acylase (PGA, penicillin amidohydrolase, EC3.5.1.11) is a heterodimeric protein (Arroyo et al., 2003). The reactions in which PGA participates and controls are hydrolysis, synthesis, and hydrolysis of esters (Duggleby et al., 1995; Shaw et al., 2000). Most of the reagents used in the preparation of 6-APA by conventional chemical synthesis methods are carcinogenic and highly toxic, causing great harm to the environment and the human body. If present in drugs and ingested, they will endanger human health. In contrast, the enzymatic hydrolysis method in which PGA is involved is gentle, more efficient, and more environmentally friendly (Pan et al., 2022). However, the free enzyme is sensitive to changes in the external environment, and the main substances in the reaction solution are difficult to separate and purify (Yang et al., 2016). With the increasing demand for beta-lactam antibiotics, PGA with high catalytic performance and easy separation is desired (Maresova et al., 2014). Immobilizing PGA on a suitable carrier to prepare immobilized PGA that is easy to store and recycle and has good stability is an effective method to avoid the drawbacks of the free PGA and meet industrial production requirements (DiCosimo et al., 2013). Immobilized PGA not only improves the recovery efficiency of PGA but also reduces production costs and simplifies the separation and purification process (Liu C. L. et al., 2022).

An inorganic carrier is characterized by high mechanical strength, strong corrosion resistance, large specific surface area, uniform pore size distribution, and low cost. Researchers graft functional groups onto inorganic materials by chemical modification, such as ─ SH, ─ CN, ─ NH2, ─ CH2 ═ CH2, ─COOH and ─CHO, and fix them through covalent binding with PGA functional groups in the PGA (Fonseca et al., 2010; Wang et al., 2019). A kind of mesoporous microsphere composed of a crosslinking agent and functional monomer is used as the carrier of immobilized PGA (Knezevic-Jugovic et al., 2016). This microsphere has many advantages such as the variety of functional groups and the number of targets that can be controlled. Crosslinked mesoporous polymer microspheres are widely used to immobilize PGA carriers and are the most widely used microspheres in the market. Eupergit C is a granular crosslinked polymer with an average pore size of 23 nm. It is the most widely used stationary PGA carrier on the market. However, some drawbacks remain, such as slow response rates and ineffective product separation (Li K. et al., 2018; Li et al., 2019). To sum up, the composite carrier combining the advantages of organic and inorganic carriers is favored, especially the combination of paramagnetic inorganic materials and organic materials, so that immobilized PGA has high thermal stability, pH stability, and excellent reusability, and can be quickly recovered by magnets (Xue et al., 2015; Li X. et al., 2018).

However, recycling after the enzymatic reaction is an important issue in the application of immobilized enzymes (Bilal et al., 2023; Chen et al., 2023; Lou et al., 2023). Using magnetic nanoparticles as carrier materials not only facilitates separation but also preserves the mobilities of PGA (Ansari and Husain, 2012; Liu et al., 2018). Magnetic nanomaterials have numerous advantages, such as high mechanical strength, large specific surface area, high mass transfer, uniform pore size distribution, facile recycling from the system, and easily modified surfaces (Yu et al., 2019a; Ling et al., 2022). There are many approaches used for the preparation of magnetic nanomaterials, such as the microemulsion method, ball-milling method, and co-precipitation and ultrasonic-assisted sol-gel methods (Akgul and Akgul, 2022; Liu W. et al., 2022; Muşat et al., 2022). However, most of these methods require complex and expensive equipment in their preparation. The preparation conditions are relatively strict, and some methods even produce nanomaterials with uneven particle sizes. Compared with traditional methods, the rapid combustion method can obtain composite material in one step, and the material properties can be controlled by controlling the reaction conditions, with its advantages including short pretreatment time, a uniform product, and convenience, (Yu et al., 2019b; Liu et al., 2019; Prakash et al., 2022; Salehizadeh et al., 2022).

The combination of the PGA and the supporter during immobilization is crucial for the reusability and activity of the immobilized PGA. Adsorption with electrostatic forces and hydrogen bonds connecting the PGA to the carrier is not commonly used because the PGA is prone to detachment (Yu et al., 2019a). When the PGA is embedded in the carrier, the diffusion rate reduces and the product and substrate are difficult to separate, which leads to a decrease in the catalytic efficiency of the immobilized PGA (Li et al., 2020). However, in the covalent cross-linking approach, PGA and carrier binding are more stable and well-behaved. The surface of nanoparticles is modified by sodium silicate hydrate, and the PGA is crosslinked with the carrier by glutaraldehyde to prepare stable immobilized PGA. Because silica has the advantages of maintaining core magnetism, chemical stability, biocompatibility, and surface modification, and glutaraldehyde is covalently cross-linked with PGA through the free amino groups of lysine residues (Liu R. J. et al., 2021; Liu C. L. et al., 2021; Pei et al., 2022), both of them are widely used in covalent crosslinking.

**FIGURE 1:**
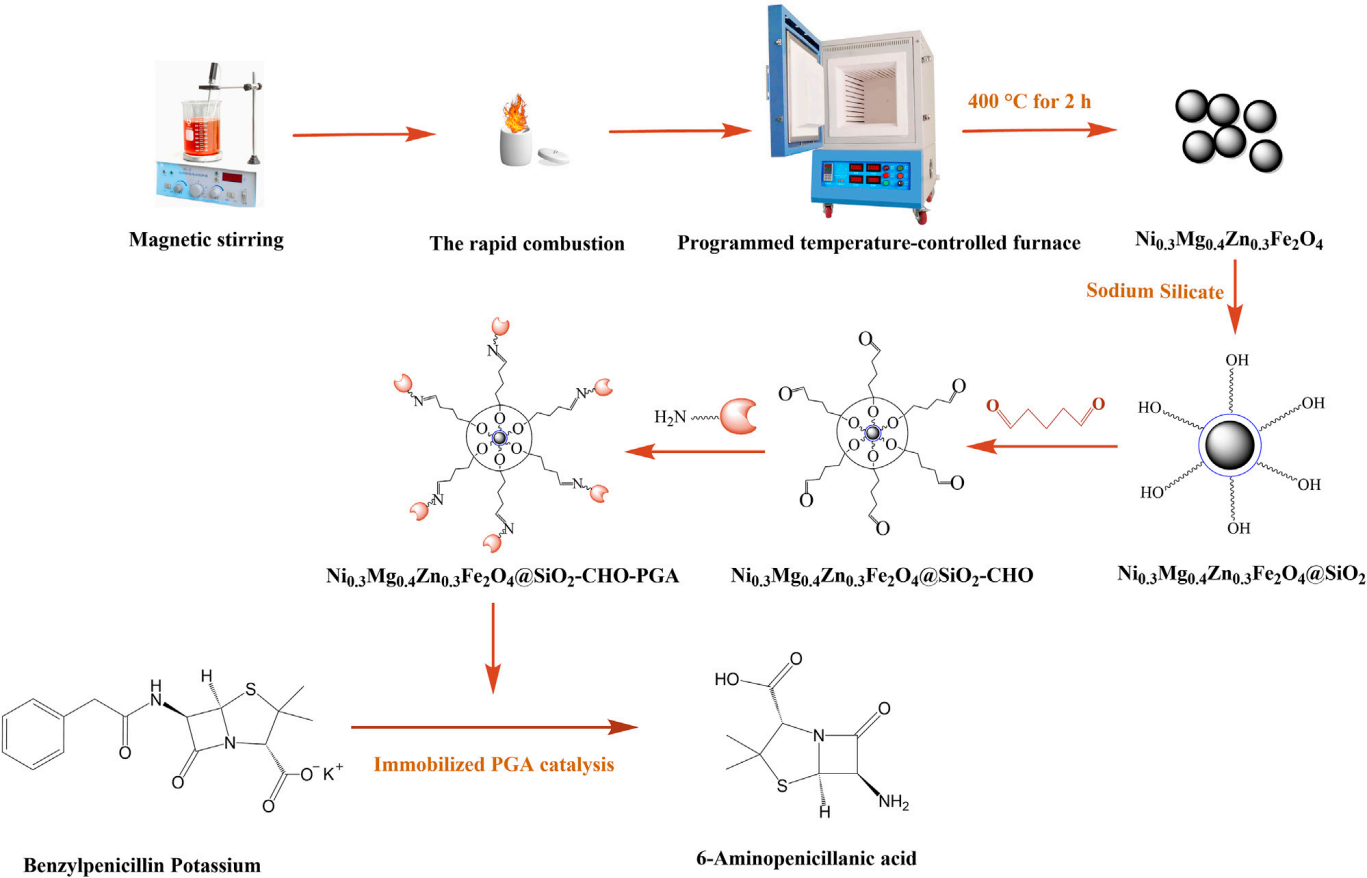
The flow chart of immobilized work.

In this project, the magnetic Ni_0.3_Mg_0.4_Zn_0.3_Fe_2_O_4_ nanoparticles were prepared by the rapid combustion method, their surface was functionalized with sodium silicate and glutaraldehyde, and the product was used as a carrier for the immobilization of PGA. The conditions for immobilizing PGA by covalent bonding were explored and optimized, and the property of the immobilized PGA was investigated. The immobilization process is shown in Figure 1.

## Experimental details

### Preparation and modification of magnetic Ni_0.3_Mg_0.4_Zn_0.3_Fe_2_O_4_ nanoparticles

The magnetic Ni_0.3_Mg_0.4_Zn_0.3_Fe_2_O_4_ nanoparticles were prepared by the rapid-combustion method according to the molar ratio 3:4:3:20 of Ni, Mg, Zn, and Fe. For the experiment, 0.78 g nickel nitrate hexahydrate, 0.92 g magnesium nitrate hexahydrate, 0.80 g zinc nitrate hexahydrate, and 7.26 g ferric nitrate non-ahydrate were put into a beaker and magnetically stirred for 30 min to make the solution dissolve completely after adding 20 mL of absolute ethanol. The solution was ignited in a crucible in an open, safe, and well-ventilated area, and the ethanol was allowed to burn out. The cooled crucible was moved into the programmed control temperature furnace at 400°C for 2 h, then magnetic Ni_0.3_Mg_0.4_Zn_0.3_Fe_2_O_4_ nanoparticles were obtained.

Next, 1.0 g magnetic Ni_0.3_Mg_0.4_Zn_0.3_Fe_2_O_4_ nanoparticles and 200 mL of distilled water were added to a round-bottomed flask, heated in a water bath at 80°C, and magnetically stirred for 3 h. When the temperature reached 80°C, sodium silicate non-ahydrate solution was slowly added and then an appropriate amount of 2.0 mol/L hydrochloric acid solution was used to keep the pH around 6. Magnetic Ni_0.3_Mg_0.4_Zn_0.3_Fe_2_O_4_@SiO_2_ nanoparticles were obtained by centrifugal washing and drying. Following this, 0.1 g Ni_0.3_Mg_0.4_Zn_0.3_Fe_2_O_4_@SiO_2_, 0.2 mL 25% glutaraldehyde, and 1 mL 0.05 mol/L PBS (pH = 7) were mixed and stirred for 2 h. Magnetic Ni_0.3_Mg_0.4_Zn_0.3_Fe_2_O_4_@SiO_2_-CHO nanoparticles were prepared.

The phase identification of the magnetic Ni_0.3_Mg_0.4_Zn_0.3_Fe_2_O_4_ nanoparticles was characterized by Rigaku D/max 2500 PC X-ray diffraction (XRD) with Cu-Kα radiation. The morphology and composition analyses were investigated with scanning electron microscopy (SEM) and transmission electron microscopy (TEM). The magnetic measurement was taken on an ADE DMS-HF-4 vibrating sample magnetometer (VSM).

### Immobilization of penicillin G acylase

For the immobilization of PGA, 0.1 g Ni_0.3_Mg_0.4_Zn_0.3_Fe_2_O_4_@SiO_2_-CHO nanoparticles were added to a mixed solution which included free penicillin G acylase (0.05, 0.10, 0.15, and 0.20 mL) and suitable PBS (pH = 8). The mixture was reacted by an oscillator at 115 r/min for different lengths of time (6, 12, 18, and 24 h). The amount of PGA in the supernatant was determined *via* Coomassie brilliant blue method. After washing the precipitate, the immobilized PGA was obtained after drying. Then, 0.1 g experimentally obtained immobilized PGA was added into 5 mL 4% penicillin K solution at the same temperature and reacted for 10 min. The concentration of 6-APA could then be measured using Bradford’s method and the activity of the immobilized PGA could be calculated. The immobilized PGA separated from the reaction solution was recycled until the activity of the immobilized PGA decreased. The immobilized rate (IR) was calculated according to the following formula (P: the amount of immobilized PGA, P_0_: the total amount of PGA added):

$$IR\left(\%\right)=\frac{P}{P_{0}}\times 100$$

### Stability and Michaelis constants of the free PGA and immobilized PGA

pH Stability: The free PGA and immobilized PGA were thoroughly mixed with different gradients PBS (pH = 6.0, 7.0, 8.0, 8.5, and 9.0). Temperature Stability: The same volume of 4% penicillin K solution was added into each test tube and reacted for a while at 20°C, 30°C, 40°C, 50°C, 55°C, and 60°C. Reaction Time: To research the effect of reaction time on PGA stability, the enzymatic reaction involving PGA was reacted for 2, 4, 6, 8, and 10 h. Michaelis Constants: The free PGA and immobilized PGA were added separately into a range of concentrations of penicillin K solution and reacted adequately under 20°C and pH = 8.

## Results and discussion

### Characterization of magnetic Ni_0.3_Mg_0.4_Zn_0.3_Fe_2_O_4_ nanoparticles

**FIGURE 2:**
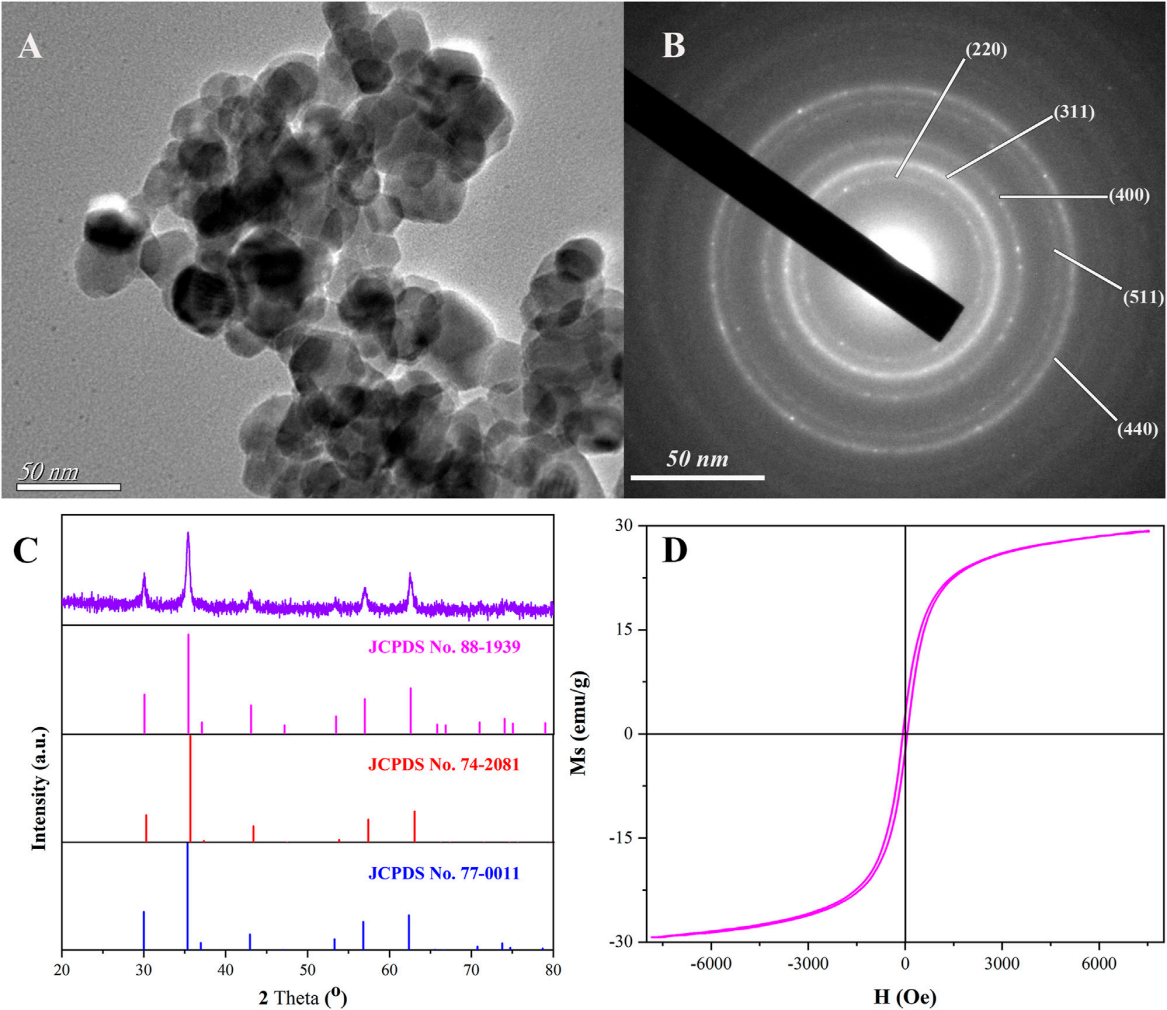
TEM image **(A)**, SAED pattern **(B)**, XRD pattern, and the corresponding standard cards **(C)** and hysteresis loops **(D)** of magnetic Ni_0.3_Mg_0.4_Zn_0.3_Fe_2_O_4_ nanoparticles calcined at 400°C for 2 h.

The characteristics of magnetic Ni_0.3_Mg_0.4_Zn_0.3_Fe_2_O_4_ nanoparticles calcined at 400°C for 2 h are shown in Figure 2. The morphology of magnetic Ni_0.3_Mg_0.4_Zn_0.3_Fe_2_O_4_ nanoparticles are displayed in Figure 2A, where the TEM image reveals that the product was spherical with a uniform distribution of particle size. The nanoparticles displayed some aggregation, possibly due to the magnetic properties of the material or the short sonication time when preparing the scanned samples. According to the statistical analysis, the average particle diameter of the prepared magnetic Ni_0.3_Mg_0.4_Zn_0.3_Fe_2_O_4_ nanoparticles was 21.7 nm. The selected area electron diffraction (Figure 2B) showed that the nanoparticles were polycrystalline. The XRD pattern of the magnetic Ni_0.3_Mg_0.4_Zn_0.3_Fe_2_O_4_ nanoparticles and the standard PDF cards of MgFe_2_O_2_ (JCPDS No. 88-1939), NiFe_2_O_2_ (JCPDS No. 74-2081), and ZnFe_2_O_2_ (JCPDS No. 77-0011) are listed in Figure 2C. According to XRD spectrogram standard PDF card, the characteristic peaks at 30.1°, 35.4°, 43.1°, 57.0°, and 62.5° were consistent with the standard card, respectively corresponding to the (220), (311), (400), (511), and (440) crystal planes. Based on the above results, it could be proved that the magnetic Ni_0.3_Mg_0.4_Zn_0.3_Fe_2_O_4_ nanoparticles had been successfully fabricated. As shown in Figure 2D, the saturation magnetization of as-prepared nanoparticles was 29.2 emu/g. Therefore, we could use the external magnetic field method to attract the immobilized materials for separation and reduce the aggregation of magnetic nanoparticles. Moreover, the magnetic Ni_0.3_Mg_0.4_Zn_0.3_Fe_2_O_4_ nanoparticles had a small renormalization and exhibited a typical soft magnetization behavior.

### Immobilization characterization of penicillin G acylase

**FIGURE 3:**
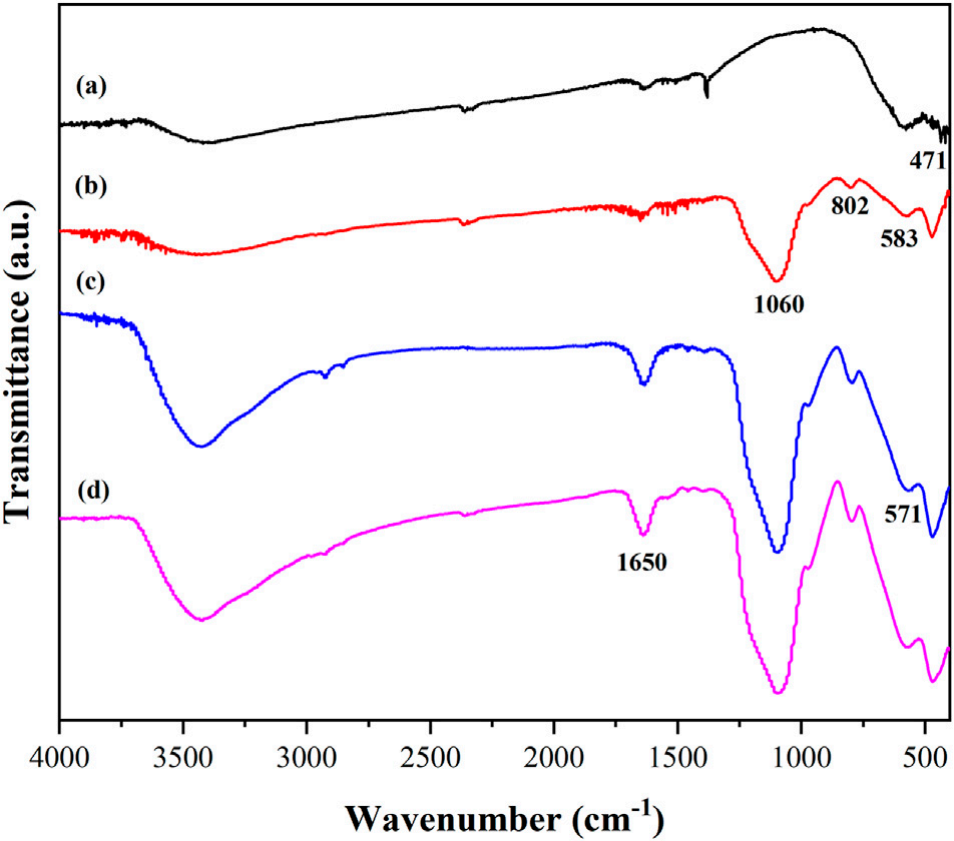
FTIR spectra of Ni_0.3_Mg_0.4_Zn_0.3_Fe_2_O_4_ nanoparticles **(A)**, Ni_0.3_Mg_0.4_Zn_0.3_Fe_2_O_4_@SiO_2_ **(B)**, Ni_0.3_Mg_0.4_Zn_0.3_Fe_2_O_4_@SiO_2_-CHO **(C)** and immobilized PGA **(D)**.

Figure 3 reveals the Fourier transform infrared spectra of the material at different stages in the PGA immobilization process. The characteristic peak at 471 cm^−1^ in Figure 3A was formed by the stretching vibration of the Fe-O bond (Yang et al., 2021), indicating that the Ni_0.3_Mg_0.4_Zn_0.3_Fe_2_O_4_ nanoparticles had been produced. However, the peak at 471 cm^−1^ in the four spectrograms in Figure 3 proved that the nanoparticle structure remains almost unchanged after encapsulation and modification. The two characteristic peaks at 802 and 583 cm^−1^ shown in Figure 3B were caused by the Si-O bond stretching vibration, and the peak of 1,060 cm^−1^ was Si-O-Si antisymmetric stretching vibration, which confirmed that the nanoparticles were successfully coated by SiO_2_ and the magnetic Ni_0.3_Mg_0.4_Zn_0.3_Fe_2_O_4_@SiO_2_ nanocomposites were obtained (Liu R. J. et al., 2021). The 571 cm^−1^ shown in Figure 3C was caused by the modification of the nanocomposites with glutaraldehyde and it was inferred that the surface of the nanocomposites was successfully connected to the aldehyde group. The characteristic peak at 1,650 cm^−1^ in Figure 3D was enhanced. This was caused by the stretching vibration of the carbon-nitrogen double bond, which was formed by the PGA and aldehyde group on the material. It could be concluded that the PGA was successfully immobilized onto the nanocomposite.

### Molecular docking studies

**FIGURE 4:**
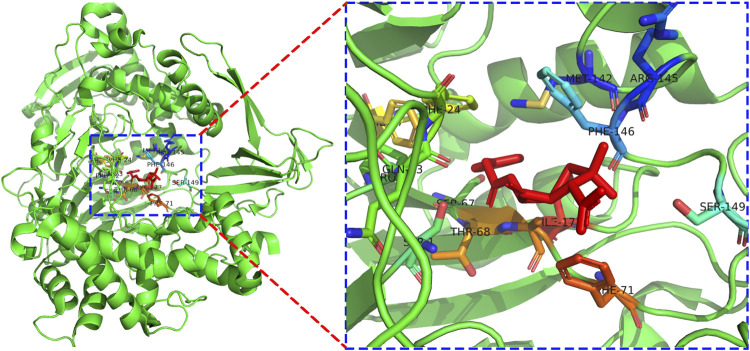
Docking pattern of PGA and penicillin G.

The docking pattern of penicillin G and PGA through the PyMol program is exhibited in Figure 4. The center of the enzyme activity in PGA resembled a rectangular pocket. Many active sites which played important roles in the catalytic action could be found from the putative site of penicillin potassium salt and PGA binding (PDB ID: 1GM8), and the substrate molecule was very close to the PGA residue due to the steric hindrance. The optimal molecular binding mode was confirmed by molecular docking and offered great value to study the conditions of immobilized PGA. For example, it demonstrated that glutaraldehyde (GA), a powerful crosslink, bound to free amino acid residues and did not impact PGA activity after crosslinking (Liu et al., 2020).

### Optimization for the immobilization of penicillin G acylase

**FIGURE 5:**
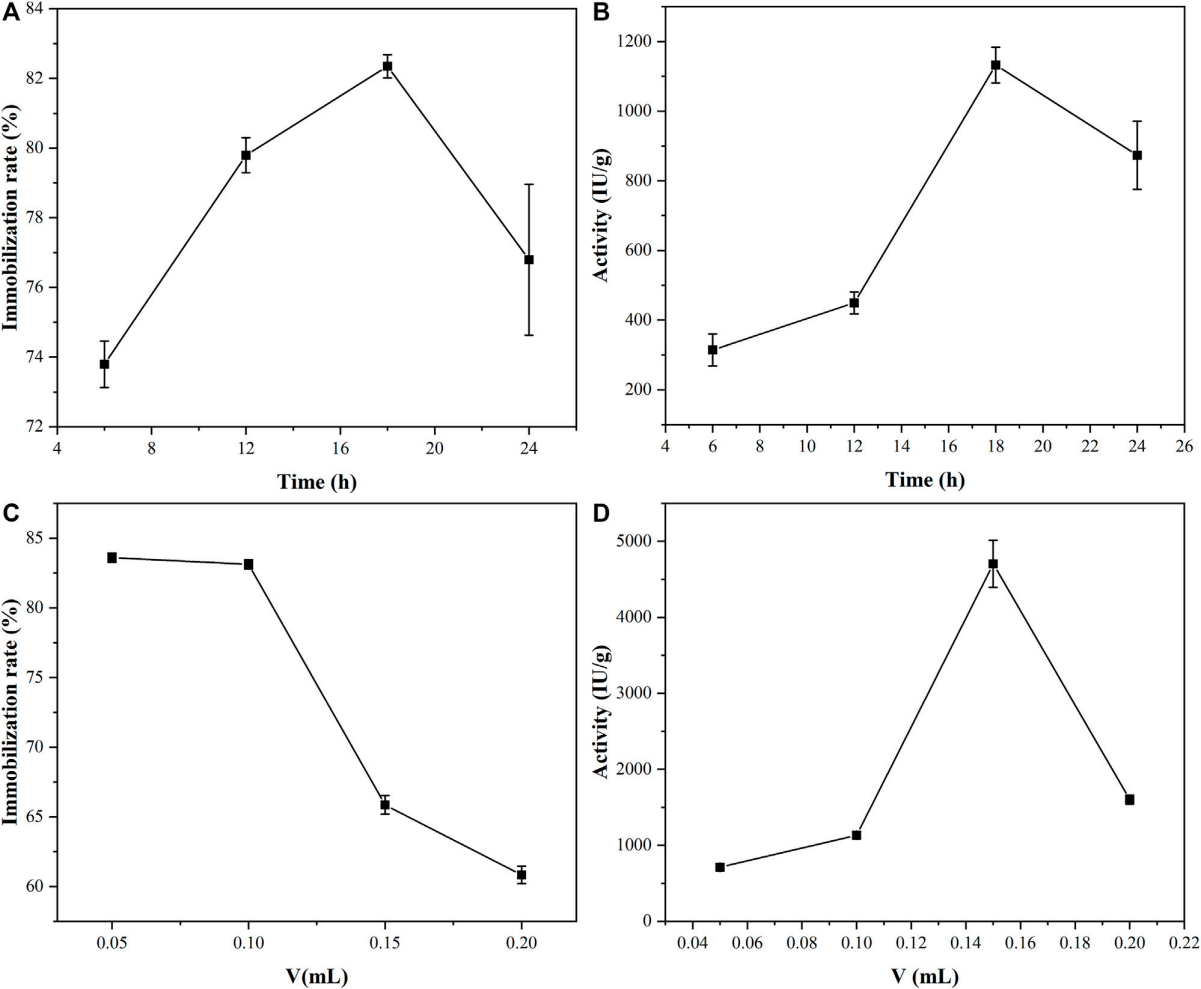
The effects of immobilization time **(A,B)** and amount of PGA **(C,D)** on immobilization rate and the activity of the immobilized PGA.

The optimal time and concentration of PGA immobilization were studied by controlling variables. The immobilization times of the PGA and the material were treated as variables to explore the alternative times for the immobilization of the PGA, while the other conditions were kept the same, and the results are demonstrated in Figures 5A, B. With the prolongation of immobilization time, the binding sites of the PGA on the material were gradually occupied, and the immobilization rate and activity of the PGA both increased. At a certain time, the binding sites of the PGA on the nanoparticles were all occupied (Liu et al., 2019), and the excess PGA was adsorbed on the material pores and covered the active site of the PGA that had been immobilized on the material, resulting in the steric hindrance of the immobilized PGA increasing and the immobilization rate and activity of the immobilized PGA decreasing. It was found that the optimal immobilization time was 18 h, so the immobilization time of 18 h was used in subsequent experiments.

The amount of PGA was taken as the variable and other conditions were unchanged. Materials and PGA were incubated together for 18 h. The results of the amount of PGA on the immobilized rate and activity were obtained, as shown in Figures 5C, D. As the amount of PGA increases, the immobilization rate of the PGA decreases, which might be related to the spatial structure of the nanoparticle and diffusion effects on the nanoparticle surface. The increased steric hindrance led to a decrease in activity.

### Stability of the free PGA and immobilized PGA

**FIGURE 6:**
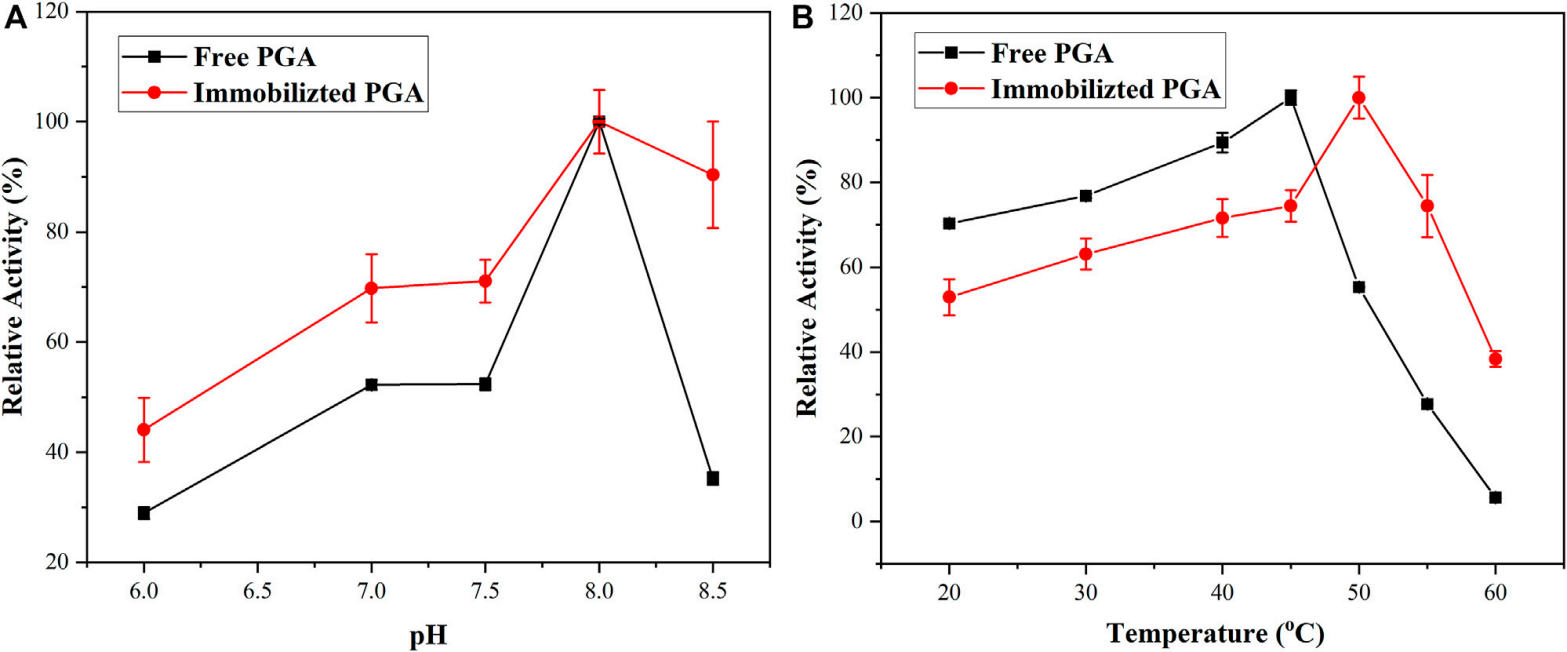
The pH **(A)** and temperature **(B)** stabilities of free PGA and immobilized PGA.

The group with the highest activity of PGA was regarded as standard and compared with the other experimental results to explore the pH stability of the PGA. As displayed in Figure 6A, with the increase in pH, the activity of the free PGA and immobilized PGA represented a trend of increasing first and then decreasing. The change in pH could affect the degree of dissociation of the necessary groups on the active center of the PGA (Liu et al., 2019), as well as the degree of dissociation of the substrate, thereby affecting the binding and catalysis of the PGA molecule to the substrate molecule. It was only at a particular pH that the dissociative states of the PGA and the substrate were most suitable for their binding and for catalyzing them to achieve the maximum relative activity. The optimal pH for both free PGA and immobilized PGA was 8.0. With the change in pH value, especially when the pH value reached 8.5, the relative activity of the free PGA dropped sharply to 35%, indicating that the pH stability of the immobilized PGA was better than that of the free PGA, and the pH application range of immobilized PGA was wide. It might be that after the PGA was immobilized, the formed diffusion effect had a certain buffering effect on pH, so the immobilized PGA was less affected by pH changes.

The thermostability of the PGA was studied using relative activity. The temperature at which the enzymatic reaction of free PGA was most complete was 45°C, as shown in Figure 6B. The immobilized PGA raised the temperature to 50°C, thereby revealing the immobilized PGA had better thermal stability. When free PGA was immobilized, the entropy and enthalpy of the protein molecule would be changed such that the thermal stability increased (Nezhad et al., 2022).

**FIGURE 7:**
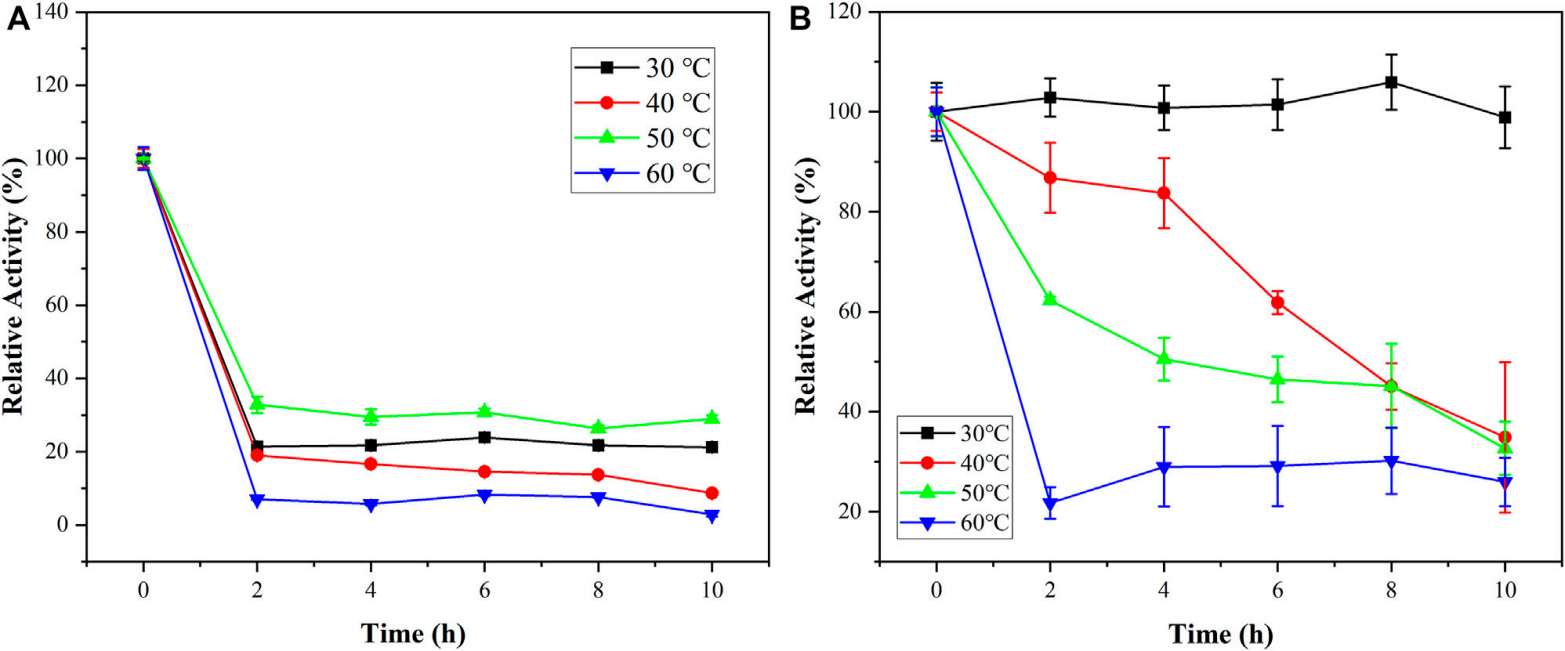
The effects of reactive time and temperature on free PGA **(A)** and immobilized PGA **(B)**.

As shown in Figure 7, the relative activities of the free PGA and immobilized PGA increased first and then decreased. But immobilized PGA had a higher optimum temperature. When the PGA was immobilized with nanoparticles, the molecular structure of PGA was stabilized, and it was less affected by temperature than free PGA. The increased temperature tolerance would benefit PGA applications in the industry.

As the enzymatic reaction time increases, the relative activity of the PGA at different temperatures was investigated to study the temporal thermostat. Figure 7 shows that the shift trends of free PGA and immobilized PGA were similar at 60°C. At 30°C, 40°C, and 50°C, the relative activities of immobilized PGA were better than those of free PGA, especially at 30°C. This indicated that the thermostability of the immobilized PGA was considerably better than that of the free PGA. The nanoparticles improved the structure of the free PGA and had some protection against the PGA active center. PGA was initially stored at 4°C and might be stored at usual temperatures below 30°C. It was also convenient for storage and use.

### Michaelis constants for free and immobilized PGA

**FIGURE 8:**
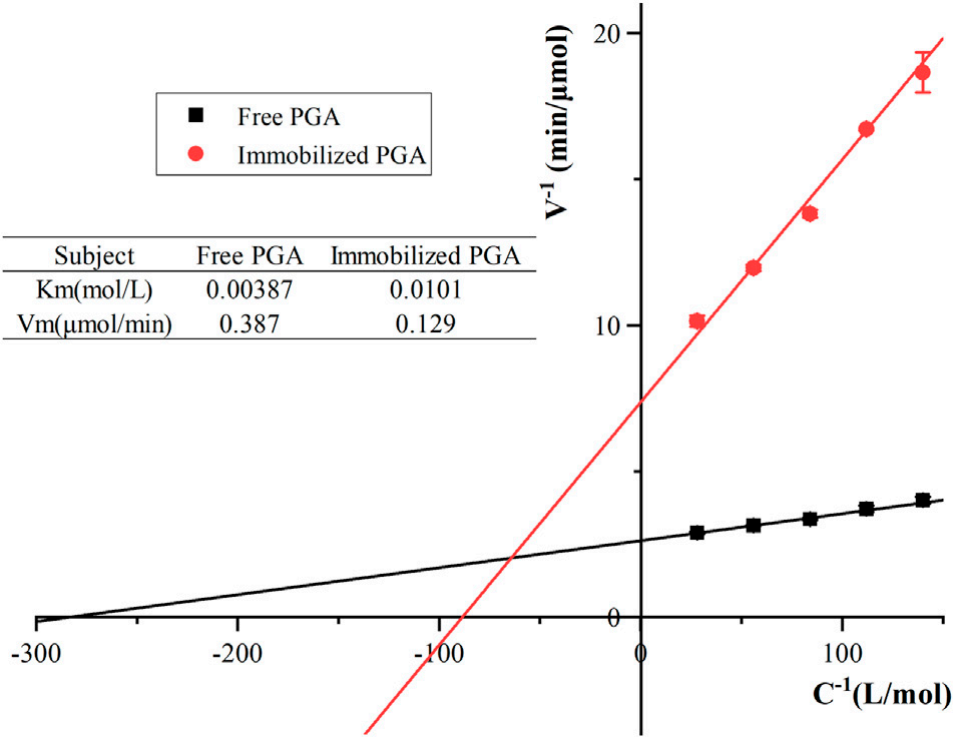
Michaelis constants of free and immobilized PGA.

The Michaelis constant (Km) represented the amount of enzyme required when the reaction rate reached half of the maximum reaction rate. It was previously one of the characteristic constants of the enzyme and related only to its properties. All other things being equal, the smaller the Km of the PGA, the larger the affinity of the PGA to the substrate. As shown in Figure 8, the Km values of the immobilized PGA and free PGA were 0.0101 and 0.00387 mol/L, while the Vmax values were 0.129 and 0.387 μmol/min. Compared with immobilized PGA, free PGA had higher substrate affinity. In the process of binding with the carrier, the spatial freedom of PGA was limited. For example, the gap between the carriers was small, so the active groups of immobilized PGA were unable to easily contact the substrate directly. It was also possible that the conformation of the active site might be damaged to different degrees, such as through shielding and extrusion (Ling et al., 2016). The different distribution of substrates in the two environments resulted from the difference between the microenvironment (the local environment of immobilized PGA) and the macroenvironment (the main solution) due to the difference in the properties of nanocomposites and substrates (Norouzian et al., 2002; Chand et al., 2015). When the substrate diffused from the macroscopic environment to the microenvironment, the concentration gradient of the substrate was formed due to the immobilized liquid film layer around PGA, which was not conducive to the flow of substrate and product. When substrate molecules arrived at the surface of PGA and transferred to the active site, the reaction efficiency would be reduced due to the existence of diffusion resistance (Ni et al., 2022).

### Repetition availability of the immobilized PGA

**FIGURE 9:**
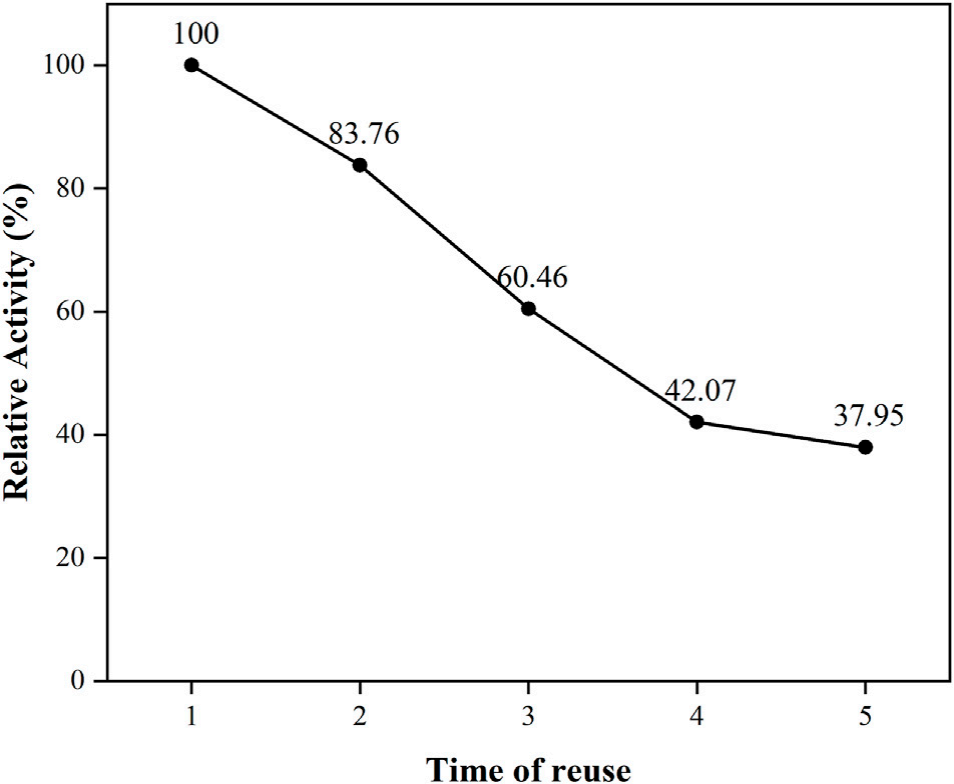
The reusability of immobilized PGA.

The free PGA was mixed with the reaction substrate and by-products after the reaction was completed, and it was difficult to separate and put back into the recycling. The immobilized PGA could be reused after magnetic separation under an external magnetic field. This could be due to the use of nanoparticles. The PGA was converted from a liquid to a solid conformation, which could be easily separated from the solution, cleaned, and put into recycling. The immobilized PGA could be reused, had superior operational stability, and saved production and application costs. The correlated activity of the immobilized PGA, which was used for the first time, was regarded as the standard, and the cycle results as displayed in Figure 9 were obtained. It could be seen that the activity of the immobilized PGA decreased uniformly with the increase in the number of uses, and after the fifth use, the enzyme activity of the immobilized PGA decreased to 37.9% of the original.

## Conclusion

The magnetic Ni_0.3_Mg_0.4_Zn0_.3_Fe_2_O_4_ nanoparticles were prepared using the rapid combustion method. Compared with free PGA, the immobilized PGA had better stability when the external pH and temperature changed, and the substrate affinity and maximum reaction rate of immobilized PGA declined. After five repeat cycles, the relative activity of the immobilized PGA decreased to 37.9%. The immobilized PGA had the advantages of repeated use, good stability, and cost saving, and it had considerable practical significance for the commercial application of PGA. These findings would facilitate further investigation into the application of PGA.

## Data Availability

The original contributions presented in the study are included in the article/supplementary material, further inquiries can be directed to the corresponding authors.
